# Catecholamine index is a simple and useful marker for bacteremic patients treated by polymyxin B hemoperfusion therapy

**DOI:** 10.1186/cc9539

**Published:** 2011-03-11

**Authors:** Y Isa, N Harayama, H Arai, T Shinjou, K Nagata, M Ueki, S Nihei, K Aibara, M Kamochi

**Affiliations:** 1 University of Occupational and Environmental Health Japan, Kitakyushu City, Fukuoka, Japan

## Introduction

Polymyxin B hemoperfusion therapy has been used for the treatment of sepsis to reduce blood endotoxin levels and a variety of inflammatory mediators. There are many reports that polymyxin B hemoperfusion therapy potentially improves circulatory dynamics and reduces mortality [1,2]. However, it is still controversial what is an important predictive factor to define the mortality. We analyzed a relationship between circulatory dynamics and mortality in our cases of polymyxin B hemoperfusion therapy.

## Methods

From January 2007 to June 2010, 69 patients who received polymyxin B hemoperfusion therapy were retrospectively reviewed. Two child cases, six cases of 24-hour death and the seven cases in whom bacteremia was not detected by blood culture test were excluded. In total, for 54 patients information including characteristics, etiological microorganisms, circulatory dynamics (catecholamine index (CAI) and mean arterial pressure (MAP)), lactate concentration and mortality was investigated. We divided the patients into survivor and nonsurvivor groups and compared these two groups. The statistical analyses were performed by unpaired *t *test.

## Results

Thirty-four patients (63.0%) survived and 20 patients (37.0%) died. Before polymyxin B hemoperfusion therapy, there were no significant differences in CAI, MAP and lactate concentration (CAI: 23.6 ± 26.5 (mean ± SD) vs. 34.0 ± 25.3, MAP: 69.7 ± 16.7 vs. 62.0 ± 16.7 mmHg, lactate: 4.0 ± 2.6 vs. 4.4 ± 3.6 mmol/l). But 2 hours after polymyxin B hemoperfusion therapy, only the CAI of the survivor group was significantly lower than in the nonsurvivor group (14.2 ± 14.1 vs. 30.4 ± 25.5; *P *< 0.01). However, MAP and lactate concentration did not show significant differences between the two groups (MAP: 80.1 ± 13.0 vs. 78.0 ± 15.4, lactate: 2.5 ± 1.3 vs. 3.6 ± 3.2). At 24 hours after polymyxin B hemoperfusion therapy, the CAI difference between the two groups was became more remarkable (6.09 ± 9.02 vs. 27.18 ± 29.31; *P *< 0.01).

## Conclusions

The CAI after polymyxin B hemoperfusion therapy was highly related to mortality, although the CAI before that therapy was not. Polymyxin B hemoperfusion therapy improve the circulatory dynamics of most sepsis patients, but the efficacy of that therapy to decreasing catecholamine is one of the important prognosis predictors for bacteremic patients.
